# Pancreatic intraductal papillary mucinous neoplasm with concomitant heterotopic pancreatic cystic neoplasia of the stomach: a case report and review of the literature

**DOI:** 10.1186/1746-1596-5-4

**Published:** 2010-01-14

**Authors:** Dimitrios Tsapralis, Alexandros Charalabopoulos, Eva Karamitopoulou, Dimitrios Schizas, Konstantinos Charalabopoulos, Theodoros Liakakos, Anastasios Macheras

**Affiliations:** 1 Third Department of Surgery, Athens University Medical School, Attikon University Hospital, Athens, Greece; 2 Department of Pathology, Athens University Medical School, Attikon University Hospital, Athens, Greece; 3 Department of Physiology, Clinical Unit, Ioannina University Medical School, Ioannina, Greece

## Abstract

A 60-year-old Caucasian male underwent a total pancreatectomy for a mixed type pancreatic intraductal papillary mucinous neoplasm (IPMN) arising in the main and secondary pancreatic ducts. During surgery, a subserosal polypoid mass was noted at the greater curvature of the gastric antrum and was enucleated. This mass was proven to be heterotopic pancreatic tissue with cystic neoplasia of the IPMN histologic subtype. Through an extensive search of the literature, we found that this is the first case ever reported with simultaneous existence of IPMN changes, in the main and secondary ducts of the orthotopic pancreas and in the heterotopic pancreatic tissue of the gastric wall.

## Introduction

Recent literature suggests either an increasing incidence of cystic neoplasms of the pancreas, or improved detection and recognition of these lesions. Historically, autopsy studies have revealed a significant prevalence of cystic lesions of the pancreas. Kimura et al [1] found cystic lesions in 24% of 300 consecutive autopsy specimens among an elderly Japanese population. The most significant recent change in the diagnosis and treatment of pancreatic cystic neoplasms is the recognition of intraductal papillary mucinous neoplasm (IPMN) as a distinct pathologic entity [2-6]. First reported in the literature by Ohashi et al [7], it was classified as a distinct entity from other mucin-producing cystic neoplasms of the pancreas by the World Health Organization (WHO) in 1996 [8]. Characteristic features of IPMN according to WHO include a tall, columnar epithelium with marked mucin production, and cystic transformation of either the main pancreatic duct or one of its side branches [8,9].

Despite the fact that IPMNs have become the second most common cause of pancreatic resections at many large centers [10], the incidence of this pathologic entity in heterotopic pancreatic tissue is extremely rare. It is not unusual to find pancreatic tissue in the stomach, duodenum, ileum, Meckel's diverticulum or at the umbilicus. Feldman and Weinberg [11] found duodenal pancreatic tissue in 13,7% of 410 necropsy specimens. Pearson [12] estimated that heterotopic pancreatic tissue could be found in as many as 2% of autopsies if it were sought carefully. In spite of the relatively common presence of heterotopic pancreas, mainly as a silent gastrointestinal malformation, a systematic review of the literature has revealed only one reported case of papillary mucinous neoplasm in gastric polypoid tumor containing heterotopic pancreatic tissue [13].

Herein, we report a case of pancreatic IPMN from the main and secondary pancreatic ducts with simultaneous existence of a gastric polypoid tumor containing heterotopic pancreatic tissue with cystic neoplasia of the same histologic subtype.

## Case report

A 60-year-old man visited his physician because of a skin discoloration suggestive of jaundice, dark urine and pale stools. The patient also reported vague epigastric pain, with onset about 6 months prior to the onset of icterus. He denied any fever or weight loss. His medical history also included hypertension, diabetes mellitus and tuberculosis. A complete blood cell count was taken, which revealed no abnormality, while the blood chemistry profile demonstrated direct hyperbilirubinemia (total bilirubin, TBIL:20 mg/dl, direct bilirubin, DBIL:16 mg/dl) and a remarkable elevation of the alkaline phosphatase (ALP) and gamma-glutamyl transpeptidase (GGT) (the levels of aspartate transaminase (AST) and alanine transaminase (ALT) were only mildly elevated). The patient was subjected to ultrasound examination of his abdomen which disclosed a cystic mass in the head of the pancreas and a dilatation of the common bile duct (1.7 cm) and intrahepatic bile ducts.

**Figure 1:**
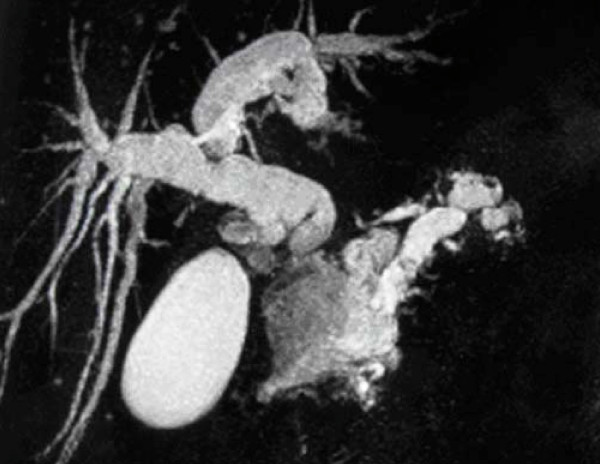
**MRCP showing pancreatic cyst in the head of the organ with dilation of the main and branch pancreatic ducts as well as of the extra- intra hepatic biliary tree**.

The patient was further evaluated with T1-weighed and T2-weighed gadolinium ((Gd)-DTPA)-enhanced MRI images and MRCP, which revealed a cystic lesion in the pancreatic head with maximum transverse diameter of 5 cm. The pancreatic cyst was in communication with a clearly dilated main pancreatic duct. In parallel with the dilatation of the main pancreatic duct along its entire course, a significant dilatation of secondary ducts (side-branches) was also documented (Figure 1). The imaging findings were compatible with the diagnosis of a diffusely distributed intraductal papillary mucinous neoplasm (IPMN) of the mixed-type variety. Moreover, the ultrasonographic finding of the dilated intra- and extra-hepatic biliary tree was confirmed, with maximum diameter of the common bile duct at about 1,7 cm. Due to the level of icterus and the coexisting dilation of the common bile duct (CBD), the patient was subsequently subjected to ERCP with simultaneous insertion of a plastic stent into the CBD. During the upper gastrointestinal endoscopy, only a mild esophagitis of the lower third of the esophagus was diagnosed, with no indication of a gastric wall abnormality reported.

After the aforementioned complete work-up of the patient, he was referred to our Surgical Department for surgical treatment. Because of the diffuse distribution of the cystic neoplasm, a total pancreatectomy, splenectomy and limited partial gastrectomy was performed. Incidentally a subserosal polypoid tumor was found at the greater curvature of the gastric antrum. Local excision of the gastric tumor was performed and it was also sent for histologic examination. On the 6^th ^postoperative day, the patient presented a biliary leak which was managed conservatively. He was discharged on the 40^th ^day, and 1 year after the operation has been disease and symptom free.

**Figure 2:**
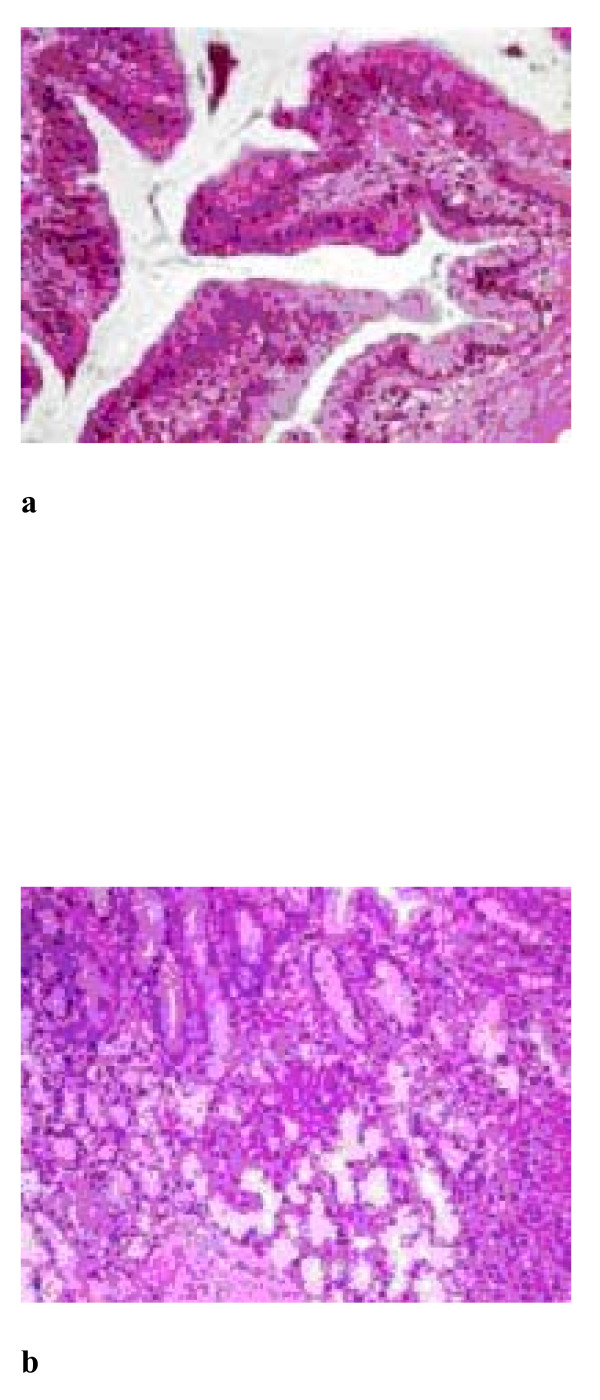
**IPMN in orthotopic pancreas**. a. Well-developed papillary projections within the duct. There is architectural and nuclear atypia. (H+E ×200), b. Budding off of clusters of neoplastic cells into the lumen (H+E ×400).

Histologic examination of the orthotopic pancreas revealed a non-invasive intraductal papillary mucinous neoplasm involving the main pancreatic duct, with prominent intraductal papillary projections (Figure 2a). The papillae were well-developed with a fibrovascular core. The neoplastic epithelial cells showed intestinal differentiation. The neoplasm exhibited significant architectural and nuclear atypia. There was budding off of clusters of neoplastic cells into the lumen, as well as, significant nuclear pleomorphism with loss of polarity and prominent nucleoli (IPMN with high grade dysplasia; figure 2b).

**Figure 3:**
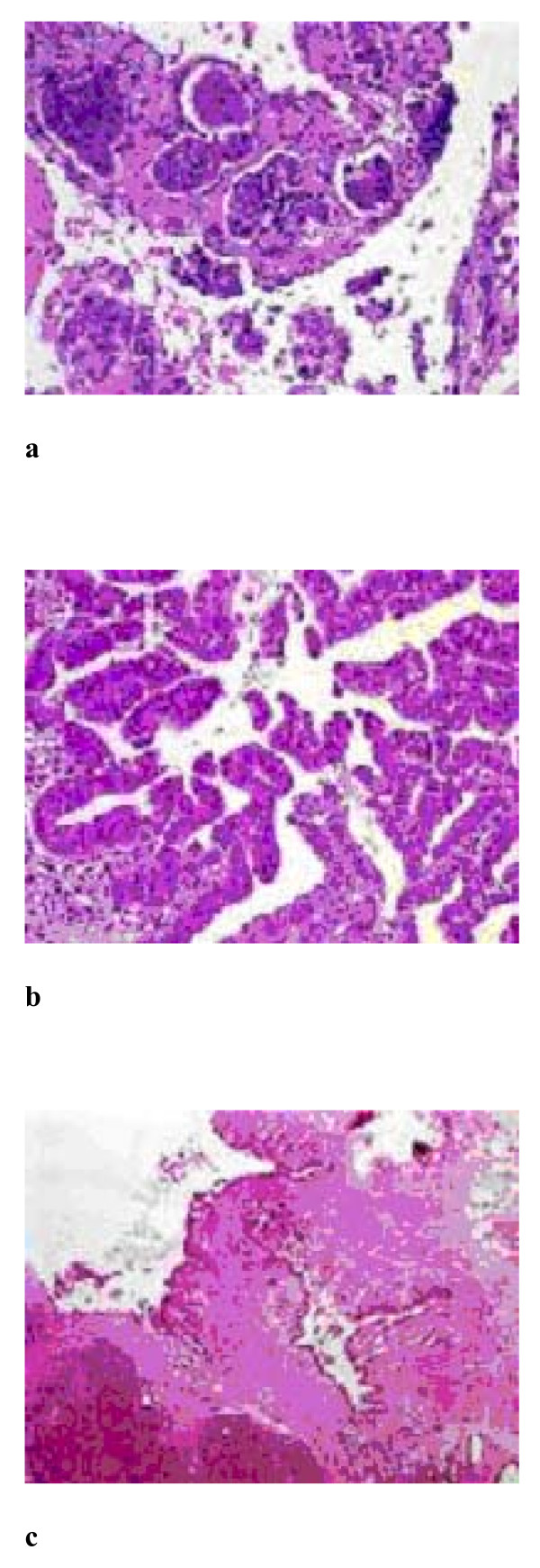
**a. Ectopic pancreatic tissue within the gastric wall (H+E ×100), b. and c**. Cystically dilated ducts containing intraluminal papillae. The papillary structures are lined by mucinous epithelium with nuclear atypia, consistent with IPMN (H+E ×400).

Additionally, a 2.5 × 2.2 × 0.9 cm measuring tissue specimen from the gastric wall was received. Macroscopic evaluation revealed a 1.5 cm white nodule with cystic spaces. Histological examination demonstrated heterotopic pancreatic tissue consisting of well-formed lobules of pancreatic acini and cystically dilated ducts containing intraluminal papillae (Figure 3a, b). The papillary structures were lined by mucinous epithelium with focal intestinal metaplasia and mild to moderate nuclear atypia (Figure 3b, c).

## Discussion

IPMNs account for 7-35% of all the cystic neoplasms of the pancreas in published surgical series [14,15]. In contrast to patients with serous cystic neoplasms (SCN) or mucinous cystic neoplasms (MCN), patients with IPMN tend to be older, with a mean age at presentation of approximately 65 years. In the differential diagnosis of IPMN MCN and pancreatic intraepithelial neoplasia (PanIN) must be included [16,17]. MCNs usually can be distinguished by the lack of pancreatic ductal structures and their characteristic ovarian-like stroma. Distinction of IPMNs from PanINs may be more difficult and has been the subject of an international consensus conference in August 2003 [17]. While IPMNs are of macroscopically visible size, PanINs are microscopic findings involving ducts less than 5 mm [17]. Moreover, IPMNs often express the mucin MUC-2, while PanINs usually express MUC-1.

Heterotopic pancreas, on the other hand, is defined as pancreatic tissue that lacks direct or vascular connection to normal pancreas [18]. In autopsy series, the prevalence of this congenital condition ranges from 0,55% to 13,7% [19]. Clinically, pancreatic heterotopia is observed in one out of 500 upper abdominal operations [20]. Pearson et al [12] reviewed 589 cases of heterotopic pancreas, and reported the frequencies of this disorder as follows: 30% in the duodenum, 25% in the stomach, 15% in the jejunum, 3% in the ileum and 6% in Meckel's diverticulum. Particularly in the stomach, heterotopic pancreatic tissue predominantly develops in males between 30 and 50 years of age. The majority of cases identified in the stomach are submucosal tumors, located in the antrum [21].

The presence of heterotopic pancreas is usually asymptomatic, but it is capable of producing symptoms, depending on its location and size [22]. Several cases have been reported in the literature presenting as gastric outlet obstruction, small bowel obstruction, upper gastrointestinal bleeding or obstructive jaundice [23-26]. Adenocarcinoma, islet cell tumors and cystic tumors have also been reported in heterotopic pancreas [19,27-29].

In the literature, there are few reported cases of malignant change of ectopic gastric pancreas [30,31]. The majority of these cases represent adenocarcinoma, while papillary mucinous neoplasia, of whatever histologic subtype, has been reported in only one case so far [13]. The present case is the first reported with the unique characteristic of simultaneous existence of IPMN (of the mixed pancreatic duct type) and IPMN or PanIN of the heterotopic gastric pancreatic tissue. Our case satisfies the minimal diagnostic criteria for tumors that arise in heterotopic pancreatic tissue initially proposed by Guillou and colleagues [29] which state that: i. the tumor must be found within or close to the ectopic pancreas, ii. direct transition between pancreatic structures and carcinoma must be observed (ie duct cell dysplasia or carcinoma in situ), iii. the non-neoplastic pancreas must comprise at least fully developed acini and ductal structures, and iv. direct extension or metastasis from an other site must be excluded.

The differential diagnosis in this case (as regards the ectopic gastric pancreatic tissue) includes low-grade intraepithelial neoplasia and small IPMN. As previously emphasized such distinction is impossible at times and currently is based on size and macroscopic appearance [32]. Since the lesion described was noted on gross inspection of the surgical specimen, we believe that the designation of intraductal papillary mucinous neoplasm would be more appropriate. Moreover, the papillary excrescences are larger than those typically seen in PanIN.

The preoperative diagnosis of heterotopic pancreas is challenging despite the advances in imaging technology. Heterotopic pancreas usually presents in upper gastrointestinal series as a well-delineated submucosal filling defect with a central indentation [20,33,34]. Endoscopically, the lesion is seen as a submucosal tumor with a central umbilication. The CT imaging of an ectopic pancreas enhances brightly as an orthotopic pancreas [29,35].

Given its clinically insidious course, heterotopic pancreas is usually an incidental finding, either intraoperativelly, or during radiographic or endoscopic examination of the upper gut. When found at the time of laparotomy (as in our case), local excision, with or without frozen section, rather than radical resection is the preferred way of treatment [25,33,36]. Potentially, however, the documentation of underlying malignancy based on the implemented frozen section analysis, sets the dilemma of performing a more radical surgical treatment in order to prevent re-operation or diagnostic difficulties.

## Competing interests

The authors declare that they have no competing interests.

## Authors' contributions

The patient was examined and operated by DT, AC, DS, TL and AM. The same authors are responsible for the post-operative care and follow up. EK performed the histopathological examination. KC was responsible for the main conception, the design and the literature review. This manuscript was drafted by DT, AC and DS, who also collected all relevant patient data, and were supervised by TL and AM. EK provided the microscopic figures and the relevant text. All authors contributed to its critical review and all approved the final draft.

## Consent

Written informed consent was obtained from the patient for publication of this case report and accompanying images. A copy of the written consent is available for review by the Editor-in-Chief of this journal.
